# Subclinical cardiovascular disease and frailty risk: the atherosclerosis risk in communities study

**DOI:** 10.1186/s12877-022-02974-z

**Published:** 2022-04-12

**Authors:** Yu Jia, Dongze Li, Jing Yu, Yi Liu, Fanghui Li, Wentao Li, Qin Zhang, Yongli Gao, Wei Zhang, Zhi Zeng, Rui Zeng, Xiaoyang Liao, Qian Zhao, Zhi Wan

**Affiliations:** 1 Department of Emergency Medicine and National Clinical Research Center for Geriatrics, Disaster Medicine Center, West China Hospital, Sichuan University West China School of Medicine, Chengdu, China; 2 West China School of Nursing, West China Hospital, Sichuan University, Chengdu, China; 3 Department of Cardiology, West China Hospital, Sichuan University, Chengdu, China; 4 Department of General Practice, International Medical Center, West China Hospital, Sichuan University, Chengdu, China

**Keywords:** Cardiovascular disease, Frailty, High sensitivity cardiac troponin T, N-terminal pro-B-type natriuretic peptide

## Abstract

**Background:**

Cardiovascular disease (CVD) is associated with a greater frailty risk, but it remains unknown if pathways that contribute to CVD are associated with the frailty risk. Thus, we aimed to investigate whether elevations in high-sensitivity cardiac troponin T (hs-cTnT) and N-terminal pro-B-type natriuretic peptide (NT-proBNP) for those without known CVD at baseline are associated with a higher frailty risk.

**Methods:**

This study used data from the Atherosclerosis Risk in Communities study. Cardiac biomarkers were measured from stored plasma samples collected at Visit 2 (1991–1993). Frailty was recorded at Visit 5 (2011–2013). Cox regression models were used to determine the association of cardiac biomarkers with frailty risk.

**Results:**

Overall, 360/5199 (6.9%) participants aged 55.1 ± 5.1 years developed frailty during a median follow-up of 21.7 years. The incidence of frailty was significantly higher in participants with hs-cTnT ≥14 ng/L (vs. < 14 ng/L: 17.9% vs. 6.7%) or NT-proBNP ≥300 pg/ml (vs. < 300 pg/ml: 19.7% vs. 6.8%) (all *P* < 0.001). Comparing higher vs. lower cut-off levels of either hs-cTnT (14 ng/l) or NT-proBNP (300 pg/ml) demonstrated a greater than two-fold higher frailty risk, with hazard ratios (HRs) of 2.13 (95% confidence interval (CI): 1.130–4.01, *P* = 0.020) and 2.61 (95% CI: 1.28–5.33, *P* = 0.008), respectively. Individuals with both elevated hs-cTnT and NT-proBNP had a higher frailty risk than those without it (HR: 4.15; 95% CI: 1.50–11.48, *P* = 0.006).

**Conclusions:**

High hs-cTnT and NT-proBNP levels are strongly associated with incident frailty in the community-dwelling population without known CVD. Subclinical cardiac damage (hs-cTnT) and/or wall strain (NT-proBNP) may be the key pathway of CVD patients developing frailty. Detection of hs-cTnT and NT-proBNP may help for early screening of high-risk frailty and providing individualised intervention.

**Trial registration:**

URL: https://www.clinicaltrials.gov; Unique identifier: NCT00005131.

**Supplementary Information:**

The online version contains supplementary material available at 10.1186/s12877-022-02974-z.

## Introduction

Frailty is a clinical syndrome with multiple causes and contributing factors [1]. It is characterised by a multisystem impairment that decreases the physiologic reserve and increases the vulnerability to stress [2]. It occurs with ageing and carries a high risk of multiple adverse health outcomes that ultimately causes hospitalisation, falls, institutionalisation, and death [3, 4].

In aging populations, cardiovascular disease (CVD) and frailty are common and often coexist [5]. Frail patients develop coronary heart disease, heart failure, and hypertension more frequently and have worse adverse outcomes than non-frail patients [6, 7]. Because the cardiovascular system fulfils several essential roles related to physical function [5], CVD is associated with a higher risk of frailty and can aggravate the severity of frailty [8, 9]. Common risk factors and related pathophysiological pathways may contribute to increased risk of both CVD and frailty [10, 11]. Moreover, early interventions, such as regular physical activity, can also decrease the risk of both frailty and CVD [12, 13]. Therefore, identifying people at high-risk of CVD may allow those who will benefit from earlier interventions to prevent frailty. However, few studies have investigated the association between subclinical CVD and frailty.

High-sensitivity cardiac troponin T (hs-cTnT) and N-terminal pro-B-type natriuretic peptide (NT-proBNP) are specific cardiac biomarkers that are highly effective for characterising subclinical CVD, such as myocardial injury (hs-cTnT) and cardiac strain (NT-proBNP) [14–16]. However, it remains unknown whether they represent two distinct and important pathways by which CVD might contribute to frailty. Therefore, this study aimed to determine whether elevated hs-cTnT and NT-proBNP are associated with an increased risk of frailty in adult participants in a community-based cohort without clinical CVD.

## Materials and methods

### Study design and population

This study used data from the Atherosclerosis Risk in Communities (ARIC) Study. Data were obtained from the public database of the National Heart, Lung, and Blood Institute Biologic Specimen and Data Repository Information Coordinating Center. Data usage was approved by the Human Ethical Committee of West China Hospital of Sichuan University. The study design and procedures of the ARIC study were established in 1987 [17]. Briefly, the ARIC is a prospective cohort study of 15,792 adults aged between 45 and 64 years from four American communities (Forsyth County, North Carolina; Jackson, Mississippi; suburban Minneapolis, Minnesota; and Washington County, Maryland). Participants were followed-up every 3 years until Visit 4 (1996–1998) and invited for Visit 5 (2011–2013) after 15 years. Physical examination and interview were carried out at each follow-up. Participants were also contacted by telephone each year to obtain information about their life conditions and hospitalisation. All participants provided written informed consent. The experimental protocol was established according to the ethical guidelines of the Helsinki Declaration and was approved by the institutional review boards at the collaborating medical centers: University of Mississippi Medical Center Institutional Review Board (Jackson Field Center); Wake Forest University Health Sciences Institutional Review Board (Forsyth County Field Center); University of Minnesota Institutional Review Board (Minnesota Field Center); and the Johns Hopkins School of Public Health Institutional Review Board (Washington County Field Center).

**Fig. 1:**
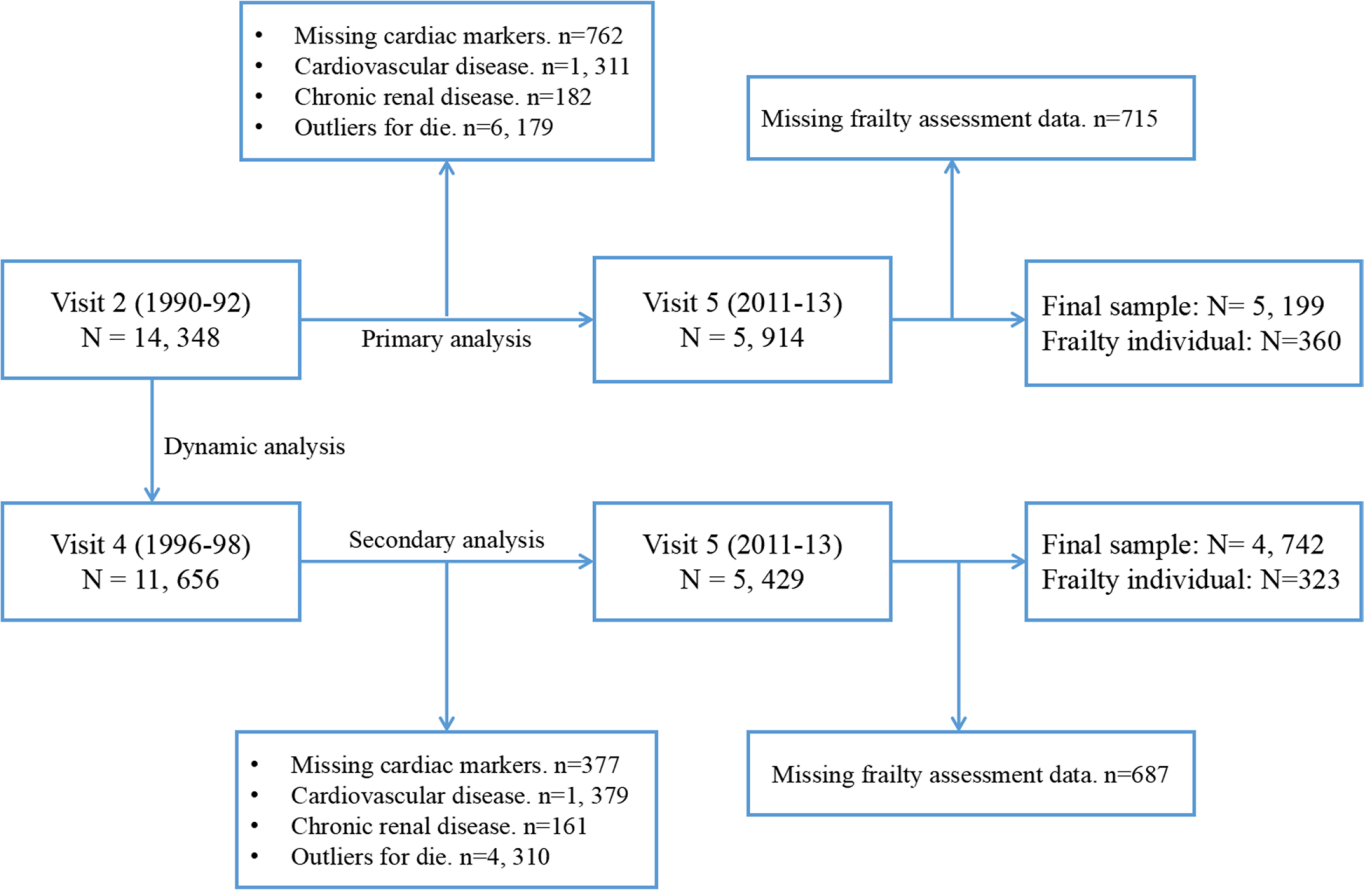
Study flow chart. hs-CTnT, high-sensitive cardiac troponin T; NT-proBNP, N-terminal pro-B-type natriuretic peptide

This study investigated the relationship of subclinical CVD, assessed by cardiac biomarkers obtained at Visit 2 (1990–1992) and Visit 4, with frailty at Visit 5. For the present study, Visit 2 was set as the baseline and there were 14,348 participants in the ARIC study. For primary analysis, we excluded participants without hs-cTnT or NT-proBNP values (*N* = 762), those with a history of CVD (*N* = 1311, including heart failure, myocardial infarction, and coronary heart disease) or chronic renal injury (*N* = 182) at baseline, those who died during follow up (*N* = 6179), and those who did not undergo frailty assessment (*N* = 715). Thus, 5199 participants were included in the primary analysis. Loss to follow-up due to mortality was of particular concern. In order to reduce the impact of mortality and decrease the possibility of reverse causality, the association of cardiac biomarkers at Visit 4 (secondary analysis) and longitudinal patterns of cardiac biomarkers from Visit 2 and Visit 4 (dynamic analysis) with risk of frailty were investigated. Finally, a total of 4727 participants were included in the secondary and dynamic analyses according to a similar exclusion standard (Fig. 1).

### Exposures: hs-cTnT, NT-proBNP, and subclinical CVD

The concentration of biomarkers was measured from stored plasma samples collected at Visit 2, using a sandwich immunoassay method on the Roche Elecsys 2010 Analyzer (Roche Diagnostics, Indianapolis, Indiana) at the University of Minnesota in 2012–2013. Samples had been stored since collection at − 70 °C. The detection limit of hs-cTnT was 5 ng/L (Elecsys Troponin T; Roche Diagnostics). The value of 1.5 ng/L was imputed for individuals with undetectable hs-cTnT. The inter-assay coefficient of variation for Hs-cTnT was 6.4% at a mean control of 29 ng/L [18]. The detection limit of NT-proBNP was 5 pg/ml. The value of 2.5 pg/ml was imputed for individuals with undetectable NT-proBNP. The inter-assay coefficient of variation for NT-proBNP was 7.4% at a mean control of 134 pg/ml [19].

An elevated hs-cTnT level was set as ≥14 ng/L, according to the 99th percentile value reported by the manufacturer, and defined as subclinical myocardial damage [20]. An elevated NTproBNP level was set as ≥100 or 300 ng/L according to previous pre-specified levels [21–23], yielding a 98% negative predictive value to exclude heart failure [24]. The predictive values of these cut-off points for CVD were also validated by previous ARIC studies [22, 25]. Thus, there were three judgment criteria for subclinical CVD in this study: hs-cTnT ≥14 ng/L, NT-proBNP ≥100, or NT-proBNP ≥300 ng/L.

### Outcomes: frailty phenotype

The frailty phenotype was classified according to the definition by the Cardiovascular Health Study and previous ARIC studies [8, 26, 27]. The frailty definition includes five component criteria: (1) Shrinking: defined as 10% unintentional weight loss between two visits or body mass index < 18.5 kg/m^2^ at the last visit. (2) Weakness: defined as sex- and BMI-specific grip strength in the lowest 20th percentile. (3) Poor endurance and energy: defined as participants who answered ‘some of the time’ or ‘most of the time’ to the statements ‘I felt everything I did was an effort’ and ‘I could not get going’ from the Centre for Epidemiological Research Depression scale. (4) Slowness: defined as the slowest 20% of the population, based on time to walk 4 m. (5) Low physical activity level: defined as the lowest 20th percentile based on the Modified Baecke questionnaire. Participants who met three or more criteria were classified as frail (even if there were missing variables); one or two criteria, pre-frail; and without these characteristics, robust. To minimize sample loss, this study used the definition of the second edition, which considers items that cannot be completed due to physiological function limitations as positive. The frailty phenotype assessment was limited to Visit 5, and frailty outcomes were defined as ≥3 criteria vs. < 3 criteria in this study.

### Assessment of covariates

Covariates were selected based on existing literature on frailty in previous ARIC studies [28–31]. The potential factors affecting frailty included age, sex, race, drinking status (never, former, current), smoking status (never, former, current), education status (less than high school, high school, college), total cholesterol (mg/dl), high-density lipoprotein (mg/dl), triglyceride (mg/dl), blood glucose (mmol/l), creatinine (mg/ml), cognitive scores, hypertension, diabetes, cancer, and chronic obstructive pulmonary disease (COPD) disease.

Alcohol use was ascertained using an interviewer-administered questionnaire. Participants were asked whether they currently consume alcohol or whether they had done previously. We defined “never drank” as participants who reported never drinking, “current drinker” as participants who reported active drinking at Visit 2, and “former drinker” as participants who had previously consumed alcohol but did not report active drinking at Visit 2 [32]. Smoking was defined in a similar manner [33]. The average systolic blood pressure and diastolic blood pressure in the sitting position was calculated with an automatic oscillographic sphygmomanometer (OMRONHEM-907XL) after sitting for 5 min. The body mass index was calculated based on height and weight measurements. Hypertension was defined as self-reported antihypertensive drug use or blood pressure ≥ 140/90 mmHg. Diabetes was defined as fasting blood glucose ≥126 mg/dl, non-fasting blood glucose ≥200 mg/dl, or self-reported antidiabetic drug use. History of CVD was defined as electrocardiogram abnormalities, self-reported history of previous coronary procedures at baseline, or any definite CVD events observed during the follow-up. The presence of COPD was based on self-reported physician diagnosis or the obstructive vital capacity measurement model, which was defined as the ratio of forced expiratory volume in 1 s /forced vital capacity < 0.70. Cancer was determined by extracting medical records and discharge codes for self-reported cases. The cognitive test included the Delayed Word Recall Test, the Word Fluency Test, and the Digit Symbol Substitution Test. Cognitive Z scores were calculated by averaging individual Z scores from three tests [34, 35].

### Statistical analysis

Parametric continuous variables were reported as the mean ± standard deviation and compared using analysis of variance. Meanwhile, non-parametric continuous variables were reported as the median (25th, 75th percentiles) and compared using the Mann-Whitney U test. Categorical variables were reported as frequencies and percentages and compared using the chi-square test.

Kaplan–Meier plots for the cumulative incidence of frailty according to elevated and low cardiac biomarker levels at Visit 2 were conducted and compared using the log-rank chi-square test. The Cox proportional hazards regression models were used to assess the relationship of the cardiac biomarkers at Visits 2 and 4 with the time to the frailty event. To further determine whether these relationships were independent of risk factors, the model was adjusted according to demographic variables, physiological variables, laboratory test results, and chronic medical conditions. To reduce sample loss caused by partly missing covariates (such as education, income, smoking, and drinking) when the Cox regression models were run, we conducted multiple imputations to simulate the missing information. Missing covariates were observed in 0.3% of participants for education and lifestyle variables, in 3.3% of participants for laboratory examination, and in 0.4% of participants for chronic conditions. The missing information was imputed simultaneously by conducting regression-based multiple imputations according to Rubin’s rules [36].

As loss to follow up due to mortality could introduce a major selective bias, additional analyses were conducted. First, Cox regression models were used to analyse the relationship between elevated cardiac biomarkers and composite events (frailty plus all-cause mortality). Further, a competing risk analysis was conducted to compare the association of elevated cardiac biomarkers with frailty and all-cause mortality, where each outcome was simultaneously modelled as a different event, and the Wald test was used to compare the parameter estimates between frailty and all-cause mortality [37].

Correlations between continuous cardiac biomarkers levels at Visit 2 and adjusted hazard ratios (HRs) for frailty events were analyzed using restricted cubic linear splines of the cardiac biomarkers with three evenly spaced knots, determined by Harrell et al.’s method [38]. In addition, after adjusting for the same confounding factors, Cox regression analysis was performed to evaluate the association of the cardiac biomarkers at Visit 2 and 4 with frailty in different subgroups of age, sex, race, hypertension, and diabetes, and their interactions were tested.

To examine the association of longitudinal patterns of midlife cardiac biomarkers (representing the change in subclinical CVD from Visit 2 to Visit 4) with frailty, the Cox regression models were conducted. Participants were divided into four groups according to two dichotomies: elevated or normal biomarkers and their change over time. The groups were as follows: (1) stable low: low cardiac biomarker at both Visits 2 and 4; (2) ascending: low cardiac biomarkers at Visit 2 and elevated cardiac biomarkers at Visit 4; (3) descending: elevated cardiac biomarkers at Visit 2 and low cardiac biomarkers at Visit 4; and (4) stable elevated: high cardiac biomarkers at Visits 2 and 4.

Considering that the two cardiac biomarkers represent different types of subclinical CVD, joint association of hs-cTnT (cut-off of 14 ng/l) and NT-proBNP (cut-off of 100 pg/ml) was evaluated by analysing their cross-categories. The population was divided into four groups: (1) low hs-cTnT plus low NT-proBNP; (2) elevated hs-cTnT plus low NT-proBNP; (3) low hs-cTnT plus elevated NT-proBNP; and (4) elevated hs-cTnT plus elevated NT-proBNP. The NT-proBNP cut-off of 300 pg/ml was not included in this analysis because only three participants with elevated hs-cTnT plus elevated NT-proBNP.

All statistical analyses were performed using SPSS version 26.0 (IBM Corp, Armonk, NY, USA) and R software 3.5.0 (Vienna, Austria). A two-tailed *P* value of < 0.05 was considered significant for all tests.

## Results

### Baseline characteristics

Among the 14,348 participants at Visit 2, 5199 participants aged 55.1 ± 5.1 years were included in this study. Among them, 360 (6.9%) and 2417 (46.5%) participants developed frailty and pre-frailty, respectively, at Visit 5, at the age of 75.7 ± 5.2 years. The baseline (1990–1992) characteristics are shown and compared in Table 1. Compared with the robust group, the frailty and pre-frailty groups were older, more likely to be female, African-Americans, have hypertension and diabetes, have lower education, and have higher concentrations of cardiovascular risk markers in midlife (Visit 2).

**Table 1:** Baseline (1990–1992) participant characteristics by frailty status at visit 5 (2011–2013)

Characteristic	Frailty	Pre-Frailty	Robust	*P*
	(*N* = 360)	(*N* = 2417)	(*N* = 2422)	
**Demographic Variables**				
Age, years	57.33 ± 5.56	55.88 ± 5.25	53.87 ± 4.65	< 0.001
Male sex	121/360 (33.6)	960/2417 (39.7)	1080/2422 (44.6)	< 0.001
African Americans	92/360 (25.6)	542/2417 (22.4)	430/2422 (17.8)	< 0.001
Education				< 0.001
Less than high school	90/360 (25.0)	366/2411 (15.2)	247/2419 (10.2)	
High school	129/360 (35.8)	799/2411 (33.1)	747/2419 (30.9)	
College	141/360 (39.2)	1246/2411 (51.7)	1425/2419 (58.9)	
Smoking				0.142
Never	172/359 (47.9)	1079/2410 (44.8)	1082/2419 (44.7)	
Former	130/359 (36.2)	913/2410 (37.9)	972/2419 (40.2)	
Current	57/359 (15.9)	418/2410 (17.3)	365/2419 (15.1)	
Drinking				< 0.001
Never	84/358 (23.5)	547/2410 (22.7)	466/2418 (19.3)	
Former	86/358 (24.0)	425/2410 (17.6)	342/2418 (14.1)	
Current	188/358 (52.5)	1438/2410 (59.7)	1610/2418 (66.6)	
**Physiological and Lab Variables**				
Body mass index, kg/m2	30.57 ± 6.58	28.11 ± 5.07	26.67 ± 4.40	< 0.001
SBP, mmHg	122.49 ± 18.12	118.85 ± 16.38	115.59 ± 16.23	< 0.001
DBP, mmHg	73.06 ± 10.13	71.97 ± 9.65	71.77 ± 9.87	< 0.001
Heart rate, /min	67.06 ± 9.47	65.1 ± 9.85	64.19 ± 9.60	< 0.001
Total cholesterol, mg/dl	5.43 ± 0.94	5.43 ± 1.00	5.32 ± 0.94	< 0.001
HDL, mg/dl	1.22 (0.96–1.5)	1.24 (1.01–1.55)	1.24 (1.01–1.6)	0.044
LDL, mg/dl	3.44 ± 0.86	3.45 ± 0.95	3.35 ± 0.88	< 0.001
Triglycerides, mg/dl	1.34 (0.95–1.9)	1.29 (0.94–1.8)	1.19 (0.86–1.7)	< 0.001
Creatinine, mg/ml	1.12 ± 0.18	1.13 ± 0.19	1.14 ± 0.18	< 0.001
Blood glucose, mmol/l	6.36 ± 2.35	6.05 ± 1.86	5.79 ± 1.37	0.001
**Chronic Medical Conditions**				
Hypertension	120/359 (33.4)	602/2413 (24.9)	431/2415 (17.8)	< 0.001
Diabetes mellitus	60/359 (16.7)	251/2410 (10.4)	129/2415 (5.3)	< 0.001
Cancer	60/354 (16.9)	408/2372 (17.2)	358/2394 (15)	0.099
COPD	2/218 (0.9)	20/1591 (1.3)	22/1658 (1.3)	0.878
Cognition Z score^a^	−0.32 ± 1.07	0.10 ± 0.95	0.22 ± 0.83	< 0.001

Values are expressed as n/N (%), mean ± SD, and median (25th, 75th). *SBP* systolic blood pressure, *DBP* diastolic blood pressure, *HDL* high density lipoprotein, *LDL* Low density lipoprotein, *COPD* chronic obstructive pulmonary disease

^a^Mean of Digit Symbol Substitution Test Z score, Word Fluency Test Z score, and Delayed Word Recall Z score

To assess whether the 5199 participants could represent the entire population at Visit 5 (*N* = 6538), we compared their baseline characteristics. The study population had similar baseline characteristics, such as age, race, and cardiovascular risk marker levels to the 1339 individuals who were excluded (*P* > 0.05); however, the study population had a slight female predominance and higher cholesterol levels (*P* < 0.05, Supplementary Table S1).

### Cardiac biomarker and frailty

There were 168 (3.2%), 909 (17.5%), and 122 (2.4%) participants with hs-cTnT ≥14 ng/L, NT-proBNP ≥100 pg/ml, and NT-proBNP ≥300 pg/ml at Visit 2, respectively. During the median follow-up of 21.7 (interquartile range: 20.0–22.3) years from Visit 2, participants with hs-cTnT ≥14 ng/L, NT-proBNP ≥100 pg/ml, or NT-proBNP ≥300 pg/ml had significant higher incidence of frailty (17.9% vs. 6.7%; 11.8% vs. 5.9%; 19.7% vs. 6.8%; *P* < 0.001 for all, respectively, Supplementary Fig. S1). Similar results were found in participants at Visit 4 (Supplementary Fig. S2).

**Fig. 2:**
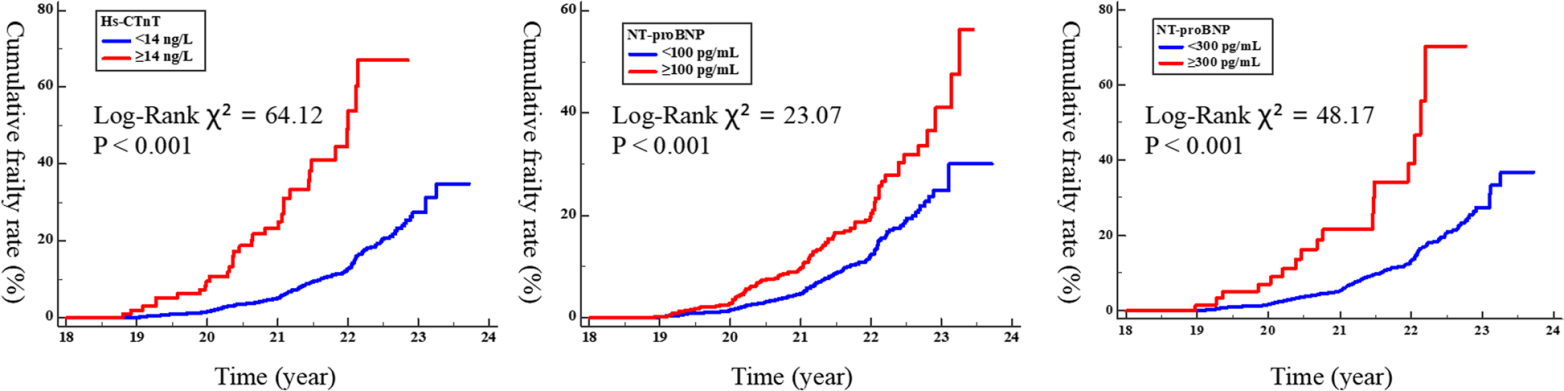
Kaplan–Meier curve for the cumulative frailty rate according to elevated and low cardiac biomarker levels. hs-CTnT, high-sensitive cardiac troponin T; NT-proBNP, N-terminal pro-B-type natriuretic peptide

In the Kaplan–Meier analysis, participants with hs-cTnT ≥14 ng/L, NT-proBNP ≥100 pg/ml, or NT-proBNP ≥300 pg/ml had a significantly higher cumulative incidence of frailty compared to participants with normal cardiac biomakers (67.5% vs. 35.1%; 56.1% vs. 30.0%; 70.1% vs. 37.8%; *P* < 0.001 for all, respectively, Fig. 2).

In Cox regression analysis, compared with participants with hs-cTnT < 14 ng/L at Visit 2, those with hs-cTnT ≥14 ng/L had an HR for frailty of 2.127 (95% confidence interval (CI): 1.129–4.010, *P* = 0.020), even after adjusting for demographic variables, physiological variables, laboratory examination, and chronic medical conditions (Table 2). Similarly, NT-proBNP ≥100 or 300 pg/ml was an independent risk factor for frailty (cut-off = 100 pg/ml, HR = 1.33, 95% CI: 1.01–1.76, *P* = 0.045; cut-off = 300 pg/ml, HR = 2.61, 95% CI: 1.28–5.33, *P* = 0.008). When models were run without multiple imputations, the results were consistent (data not shown). However, NT-proBNP ≥300 pg/ml at Visit 4 was not independently associated with incident frailty (*P* > 0.05, Supplementary Table S4).

**Table 2:** Adjusted HRs (95% CI) for the association of baseline (1991–1993) hs-cTnT and NT-proBNP with incident frailty

Variable	Hs-CTnT (≥14 vs. < 14 ng/L)		NT-proBNP (≥100 vs. < 100 pg/mL)		NT-proBNP (≥300 vs. < 300 pg/mL)	
	HR (95% CI)	*P*	HR (95% CI)	*P*	HR (95% CI)	*P*
Unadjusted	2.569 (1.532–4.310)	< 0.001	1.903 (1.518–2.386)	< 0.001	2.676 (1.505–4.760)	< 0.001
Model 1	2.156 (1.254–3.708)	0.005	1.643 (1.282–2.105)	< 0.001	2.233 (1.244–4.011)	0.007
Model 2	2.127 (1.129–4.010)	0.020	1.330 (1.007–1.756)	0.045	2.614 (1.283–5.327)	0.008

Model 1: adjusted by age, sex, center-race, education (<high school, high school, or > high school), smoking (never, former, current), drinking (never, former, current), body mass index, systolic blood pressure, heart rate, total cholesterol, triglycerides

Model 2: adjusted by model 1 plus cognition Z score, hypertension, diabetes, and cancer

*hs-CTnT* high-sensitive cardiac troponin T, *NT-proBNP* N-terminal pro-B-type natriuretic peptide, *HR* hazard ratio, *CI* confidence interval

Participants with elevated hs-cTnT (≥14 ng/L) or NT-proBNP (≥300 pg/ml) levels had more than a five-fold increased risk of frailty combined with mortality (*P* < 0.001, Supplementary Table S2). However, in the competing risk analysis, the association of elevated hs-cTnT (≥14 ng/L) or NT-proBNP (≥300 pg/ml) at Visit 2 with frailty showed no change for all-cause mortality (*P* = 0.647; *P* = 0.781; respectively).

**Fig. 3:**
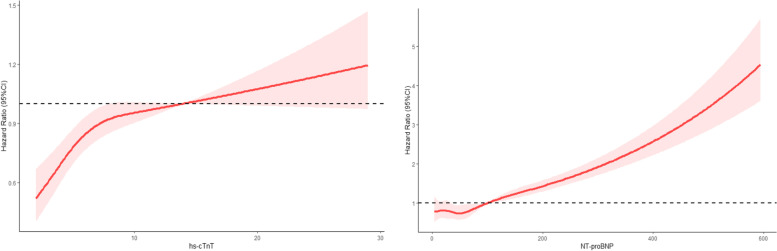
Adjusted hazard ratios (95% CI) of frailty events by linear splines of high-sensitive cardiac troponin T (hs-CTnT) and N-terminal pro-B-type natriuretic peptide (NT-proBNP) with three same spacing knots. The solid line indicates the point estimate, and the shaded area is the 95% CI. Models were adjusted by age, sex, center-race

In the restricted cubic spline models, the adjusted HR for incident frailty increased linearly with an increase in hs-cTnT and NT-proBNP levels at Visit 2 (Fig. 3). The relationship between hs-cTnT and frailty was curvilinear, with the appearance of a plateau for values over 14 ng/L. In contrast, the relationship between NT-proBNP and frailty was rectilinear. Regardless of the cut-off point of 100 or 300 pg/ml, the curve had a high slope, indicating that there was no unique threshold for NT-proBNP (Fig. 3).

At Visit 2 (Supplementary Table S3) and Visit 4 (Supplementary Table S4), subgroup analysis showed that the hs-cTnT and NT-proBNP levels were also associated with frailty events in different subgroups of age, sex, race, and chronic condition (P for interaction > 0.05).

#### Longitudinal patterns of cardiac biomarkers and frailty

From Visit 2 to Visit 4, participants with stable low levels of cardiac biomarkers had significant lower incidence than that of participants with ascending and stable elevated levels of cardiac biomarkers (*P* < 0.05, Supplementary Fig. S3). After adjusting for multiple confounding factors, ascending and stable elevated levels of cardiac biomarkers were independently associated with incident frailty (Table 3).

**Table 3:** Multivariate Cox regression analysis of longitudinal patterns of cardiac biomarker for frailty events according to the “low” versus “elevated” dichotomization

Variable	N (%)	Multivariate Cox regression			
		HR (95% CI) for model 1	*P*	HR (95% CI) for model 2	*P*
**Hs-CTnT (cut-off: 14 ng/l)**			0.002		0.025
Stable low	4270 (90.0)	REF.		REF.	
Ascending	320 (6.8)	1.89 (1.13–3.15)	0.015	2.18 (1.18–4.05)	0.013
Descending	46 (1.0)	0.43 (0.35–3.52)	0.432	1.07 (0.24–8.22)	0.946
Stable elevated	106 (2.2)	3.03 (1.45–6.31)	0.003	2.70 (1.00–7.26)	0.048
**NT-proBNP (cut-off: 100 pg/ml)**			0.001		0.006
Stable low	3186 (67.2)	REF.		REF.	
Ascending	751 (15.8)	1.33 (0.98–1.81)	0.067	1.55 (1.05–2.29)	0.027
Descending	231 (4.9)	1.26 (0.77–2.06)	0.364	1.47 (0.81–2.67)	0.206
Stable elevated	574 (12.1)	1.88 (1.37–2.58)	< 0.001	1.97 (1.30–3.00)	0.001
**NT-proBNP (cut-off: 300 pg/ml)**			0.030		0.083
Stable low	4380 (92.8)	REF.		REF.	
Ascending	248 (5.2)	1.27 (0.68–2.37)	0.451	1.54 (0.72–3.29)	0.266
Descending	62 (1.4)	2.21 (0.81–6.04)	0.122	2.86 (0.62–13.17)	0.177
Stable elevated	52 (1.0)	3.52 (1.31–9.46)	0.013	3.49 (1.21–10.02)	0.020

Model 1: adjusted by age, sex, center-race, education (<high school, high school, or > high school), smoking (never, former, current), drinking (never, former, current), body mass index, systolic blood pressure, heart rate, total cholesterol, triglycerides

Model 2: adjusted by model 1 plus cognition Z score, hypertension, diabetes, and cancer

*hs-CTnT* high-sensitive cardiac troponin T, *NT-proBNP* N-terminal pro-B-type natriuretic peptide, *HR* hazard ratio, *CI* confidence interval

### Joint association of hs-cTnT and NT-proBNP

**Fig. 4:**
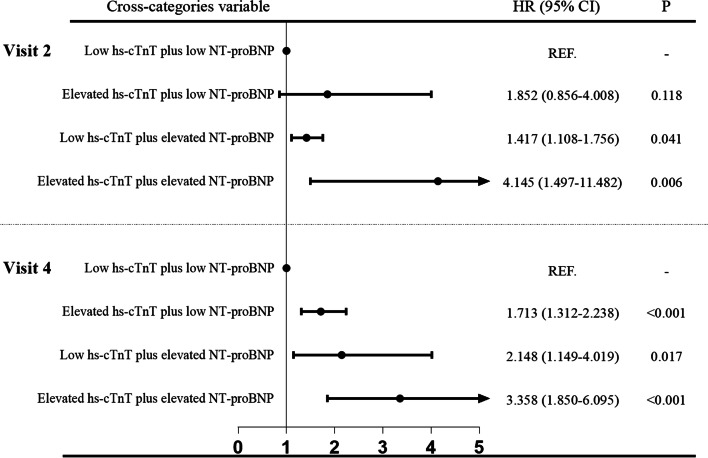
Adjusted HRs (95% CIs) for the association of cross-categories of hs-cTnT and NT-proBNP both at visit 2 and and visit 4 with frailty events. Multivariate Cox regression analysis between cardiac biomarker and frailty adjusted by age, sex, center-race, education, current smoking, current drinking, body mass index, systolic blood pressure, heart rate, total cholesterol, triglycerides, cognition Z score, hypertension, diabetes, and cancer

Data from Visit 2 were entered into a multivariate Cox regression model for the correlation analysis between joint association of cardiac biomarkers and frailty. The results showed that individuals with elevation of both hs-cTnT (≥14 ng/L) and NT-proBNP (≥100 pg/ml) had a higher risk for frailty than those with low cardiac biomarker levels (HR: 4.15, 95% CI: 1.50–11.48, *P* = 0.006). In addition, the HR for elevated NT-proBNP alone was 1.42 (95% CI: 1.11–1.76, *P* = 0.041). However, elevated hs-cTnT alone did not evidently confer an increased frailty risk (Fig. 4). In Visit 4, participants with any elevation in hs-cTnT (≥14 ng/L) or NT-proBNP (≥100 pg/ml) categories had a significantly higher risk of frailty than individuals with low cardiac biomarker levels (Fig. 4).

## Discussion

CVD is associated with frailty, but it remains unknown whether pathways that contribute to CVD are also associated with the frailty risk. The present study demonstrated that the biomarkers of subclinical myocardial injury (hs-cTnT) and cardiac wall strain (NT-proBNP) are strongly and independently associated with incident frailty in the community-dwelling population. The subgroup analysis results showed that the relationship between cardiac biomarkers and frailty was stronger in participants aged < 55 years at baseline. This is probably due to the loss to follow-up of older participants with higher biomarkers. Given that we used a competing risk model and found that the association of elevated hs-cTnT or NT-proBNP levels with frailty was independent of all-cause mortality; these findings suggest that biomarkers of cardiac injury and wall strain could be important independent risk factors for frailty and may help identify novel, independent pathways linking CVD to frailty among the general population with no history of clinical CVD.

Prior studies have shown that cTnT and NT-proBNP are additive in predicting the risk of mortality and bleeding events in the community [30, 39]. We suspected that the combination of hs-cTnT and NT-proBNP can increase the effectiveness of identifying frailty risk because hs-cTnT and NT-proBNP reflect different sub-health states of cardiac function. The results showed that individuals with both elevated hs-cTnT and NT-proBNP levels had a three-fold higher risk of frailty than those with low cardiac biomarker levels, and this risk was higher than that of individuals with only one marker. Further, to investigate the association of dynamic changes in cardiac biomarkers with frailty events, longitudinal analyses were conducted. The results showed that ascending and stable elevated levels of cardiac biomarkers were independent predictors for further frailty when compared to stable low levels of cardiac biomarkers. This result suggests the efficacy of cardiac biomarkers in the dynamic assessment of frailty risk. Importantly, the risk of frailty was reduced after the reversal of subclinical CVD emphasized that intervention measures for preclinical CVD is of great importance in preventing the occurrence of frailty events.

Previous studies have confirmed that frailty and CVD are mutually causal and that one promotes the other. The extent of subclinical CVD has been shown to be a strong predictor of future clinical CVD [40, 41]. Our research adds further evidence that even in those without clinically manifested CVD, the underlying abnormalities in cardiovascular function are independently related to the frailty status. This conclusion emphasizes that early identification of those at high risk of frailty according to the subclinical CVD status may help provide timely treatment and cardiac rehabilitation. This will potentially reduce the burden of frailty in the high-risk, vulnerable population and improve outcomes. In addition, as frailty is a consequence of biological aging, exploring the additional effects of subclinical CVD on aging will be useful.

Our study has some limitations. The results of interaction by age and sex with cardiac biomarkers could be a chance finding and should thus be further investigated, especially considering the contrary results according to different cut-off values of NT-proBNP in subgroup analysis. Although this is a cohort study, the baseline data of frailty cannot be captured, and, thus, the observed association may not be causal. In addition, it is unknown whether the diagnosis of frailty can be maintained when assessed according to other proposed criteria. Despite multiple imputations to missing covariate, residual bias from selective attrition is possible because missing frailty assessments may not be random, thus, it is difficult to obtain accurate HR. However, this is going to be an almost unavoidable issue in most studies of ageing. Although the results of this study were adjusted by multiple confounding factors, there may still be potential variables that were not included. Additional factors affecting the association of these biomarkers with frailty, such as biological variations in biomarker sampling and changes of biomarkers over time, were also not analysed in this study. Finally, the generalisability of our findings is unknown for younger samples or samples outside the general community environment. Given that subclinical CVD is an independent risk factor for frailty and that cardiac biomarkers are useful indicators for predicting frailty, future studies need to further confirm whether targeted interventions to reduce the concentration of biomarkers can reduce the incidence of frailty for individuals without known CVD.

In conclusion, participants with subclinical CVD, defined as elevations of biomarkers of cardiac damage (cTnT) and/or wall strain (NT-proBNP), had a significantly higher frailty risk, and this may be the key pathway of CVD patients developing frailty. Hs-cTnT and NT-proBNP may help predict frailty in the general population. Further, the combination of these two cardiac biomarkers improved the accuracy of prediction. The high-risk population should be provided individualised management, appropriate intervention, and specific treatment in the earliest possible time to help reduce the risk of frailty and improve the quality of life and patient outcomes.

## Supplementary Information

**Additional file 1.**

**Additional file 2.**

**Additional file 3.**

**Additional file 4.**

## Data Availability

Data and materials can be obtained from the website: https://biolincc.nhlbi.nih.gov/home/
