# Supplementary material for: Subclinical cardiovascular disease and frailty risk: the atherosclerosis risk in communities study
Source: BMC Geriatr. 2022 Apr 12;22:321. doi: 10.1186/s12877-022-02974-z (PMC9006603; doi:10.1186/s12877-022-02974-z)
Supplement: Supplementary file 1 — Additional file 1. [file 12877_2022_2974_MOESM1_ESM.docx]

Supplementary Table S1. Baseline (1990-1992) characteristics of the population participating in the Visit 5 according to the classification of inclusion and exclusion.

| Characteristic | | Included participants | Excluded participants | P |
| --- | --- | --- | --- | --- |
|  |  | (N=5199) | (N=1339) |  |
| **Demographic Variables** | |  |  |  |
|  | Age, years | 55.05±5.14 | 55.3±5.19 | 0.110 |
|  | Male sex | 2161/5199 (41.6) | 598/1339 (44.7) | 0.041 |
|  | African Americans | 1064/5199 (20.5) | 298/1339 (22.3) | 0.150 |
|  | Education |  |  | 0.018 |
|  | Less than high school | 703/5190 (13.5) | 221/1337 (16.5) |  |
|  | High school | 1675/5190 (32.3) | 407/1337 (30.4) |  |
|  | College | 2812/5190 (54.2) | 709/1337 (53.0) |  |
|  | Smoking |  |  | 0.287 |
|  | Never | 2333/5188 (45) | 540/1150 (47) |  |
|  | Former | 2015/5188 (38.8) | 443/1150 (38.5) |  |
|  | Current | 840/5188 (16.2) | 167/1150 (14.5) |  |
|  | Drinking |  |  | 0.186 |
|  | Never | 1097/5186 (21.2) | 269/1150 (23.4) |  |
|  | Former | 853/5186 (16.4) | 194/1150 (16.9) |  |
|  | Current | 3236/5186 (62.4) | 687/1150 (59.7) |  |
| **Physiological and Lab Variables** | |  |  |  |
|  | Body mass index, kg/m2 | 27.61±5.01 | 27.51±5.13 | 0.527 |
|  | SBP, mmHg | 117.58±16.56 | 118.24±17.33 | 0.228 |
|  | DBP, mmHg | 71.95±9.79 | 72.14±10.04 | 0.549 |
|  | Heart rate, /min | 64.81±9.74 | 64.35±9.95 | 0.149 |
|  | Total cholesterol, mg/dl | 5.38±0.97 | 5.29±0.93 | 0.005 |
|  | HDL, mg/dl | 1.32±0.43 | 1.27±0.42 | 0.001 |
|  | LDL, mg/dl | 3.4±0.91 | 3.36±0.86 | 0.126 |
|  | Triglycerides, mg/dl | 1.46±0.78 | 1.49±0.68 | 0.239 |
|  | Creatinine, mg/ml | 1.13±0.18 | 1.14±0.17 | 0.035 |
|  | Blood glucose,mmol/l | 5.95±1.70 | 5.91±1.23 | 0.428 |
| **Chronic Medical Conditions** | |  |  |  |
|  | Hypertension | 1153/5187 (22.2) | 275/1150 (23.9) | 0.216 |
|  | diabetes mellitus | 440/5184 (8.5) | 111/1134 (9.8) | 0.160 |
|  | Cancer | 44/3467 (1.3) | 18/894 (2) | 0.094 |
|  | COPD | 826/5120 (16.1) | 203/1141 (17.8) | 0.172 |
|  | Cognition Z score* | 0.01±1 | -0.05±0.98 | 0.077 |

Values are expressed as n/N (%), mean ± SD, and median (25th, 75th). SBP, systolic blood pressure; DBP, diastolic blood pressure; HDL, high density lipoprotein; LDL, Low density lipoprotein; COPD, chronic obstructive pulmonary disease; *Mean of Digit Symbol Substitution Test Z score, Word Fluency Test Z score, and Delayed Word Recall Z score.

Supplementary Table S2. Association of baseline (1990-1992) hs-cTnT and NT-proBNP with combined outcome (frailty plus mortality).

| Cardiac biomarkers | Events/N (%) | Unadjusted | |  | multivariate adjusted | |
| --- | --- | --- | --- | --- | --- | --- |
|  |  | HR (95% CI) | P |  | HR (95% CI) | P |
| **Hs-CTnT** |  |  |  |  |  |  |
| <14 ng/L | 5460/10753 (50.8) | REF. | - |  | REF. | - |
| ≥14ng/L) | 610/676 (90.2) | 9.24 (7.16-11.91) | <0.001 |  | 6.79 (4.92-9.36) | <0.001 |
| **NT-proBNP** |  |  |  |  |  |  |
| <100 pg/mL | 4118/8597 (47.9) | REF. | - |  | REF. | - |
| ≥100 pg/mL | 1952/2832 (68.9) | 2.21 (2.04-2.40) | <0.001 |  | 2.17 (1.95-2.42) | <0.001 |
| **NT-proBNP** |  |  |  |  |  |  |
| <300 pg/mL | 5578/10880 (51.3) | REF. | - |  | REF. | - |
| ≥300 pg/mL | 492/549 (89.6) | 8.63 (6.56-11.35) | <0.001 |  | 6.50 (4.85-8.72) | <0.001 |

Multivariate Cox regression analysis between cardiac biomarker and combined outcome adjusted by age, sex, center-race, education (<high school, high school, or >high school), smoking (never, former, current), drinking (never, former, current), body mass index, systolic blood pressure, heart rate, total cholesterol, triglycerides, cognition Z score, hypertension, diabetes, and cancer. hs-CTnT, high-sensitive cardiac troponin T; NT-proBNP, N-terminal pro-B-type natriuretic peptide. HR, hazard ratio; CI, confidence interval.

Supplementary Table S3. Adjusted HRs (95% CIs) for the association of baseline (1990-1992) hs-cTnT and NT-proBNP with incident frailty in different subgroups.

| Variable | Hs-CTnT (≥14 vs. <14 ng/L) | | |  | NT-proBNP (≥100 vs. <100 pg/mL) | | |  | NT-proBNP (≥300 vs. <300 pg/mL) | | |
| --- | --- | --- | --- | --- | --- | --- | --- | --- | --- | --- | --- |
|  | HR (95% CI) | P | P for interaction |  | HR (95% CI) | P | P for interaction |  | HR (95% CI) | P | P for interaction |
| **Age*** |  |  | 0.658 |  |  |  | 0.575 |  |  |  | 0.018 |
| <55 | 3.58 (1.03-12.44) | 0.045 |  |  | 1.86 (1.13-3.04) | 0.013 |  |  | 4.89 (1.49-15.97) | 0.009 |  |
| ≥55 | 1.96 (1.19-3.96) | 0.038 |  |  | 1.21 (1.08-1.71) | 0.026 |  |  | 1.31 (0.53-3.23) | 0.557 |  |
| **Gender** |  |  | 0.469 |  |  |  | 0.001 |  |  |  | 0.568 |
| Male | 2.45 (1.16-5.14) | 0.018 |  |  | 2.06 (1.19-3.57) | 0.009 |  |  | 1.01 (0.22-4.61) | 0.984 |  |
| Female | 2.37 (0.77-7.34) | 0.132 |  |  | 1.14 (0.83-1.58) | 0.401 |  |  | 2.44 (1.08-5.52) | 0.031 |  |
| **Race** |  |  | 0.093 |  |  |  | 0.923 |  |  |  | 0.303 |
| White | 2.11 (0.65-6.80) | 0.208 |  |  | 1.44 (1.07-1.94) | 0.016 |  |  | 2.12 (0.94-4.80) | 0.069 |  |
| Black | 2.54 (1.22-5.28) | 0.012 |  |  | 1.54 (0.85-2.77) | 0.152 |  |  | 5.33 (1.22-23.14) | 0.025 |  |
| **Hypertension** |  |  | 0.050 |  |  |  | 0.672 |  |  |  | 0.403 |
| No | 2.23 (1.01-4.91) | 0.046 |  |  | 1.79 (1.19-2.71) | 0.006 |  |  | 3.40 (1.45-7.92) | 0.005 |  |
| Yes | 0.89 (0.400-2.01) | 0.784 |  |  | 1.74 (1.02-2.99) | 0.042 |  |  | 1.35 (0.41-4.38) | 0.618 |  |
| **Diabetes** |  |  | 0.928 |  |  |  | 0.600 |  |  |  | 0.202 |
| No | 2.19 (1.36-4.99) | 0.033 |  |  | 1.99 (1.41-2.82) | <0.001 |  |  | 2.87 (1.38-5.94) | 0.004 |  |
| Yes | 1.46 (0.19-11.24) | 0.715 |  |  | 1.57 (0.63-3.89) | 0.330 |  |  | 1.33 (0.15-11.92) | 0.795 |  |

*The population was classified according to the median of age. Multivariate Cox regression analysis between cardiac biomarker and frailty adjusted by age, sex, center-race, education (<high school, high school, or >high school), smoking (never, former, current), drinking (never, former, current), body mass index, systolic blood pressure, heart rate, total cholesterol, triglycerides, cognition Z score, hypertension, diabetes, and cancer. hs-CTnT, high-sensitive cardiac troponin T; NT-proBNP, N-terminal pro-B-type natriuretic peptide. HR, hazard ratio; CI, confidence interval.

Supplementary Table S4. Adjusted HRs (95% CIs) for the association of hs-cTnT and NT-proBNP at visit 4 (1996-1998) with incident frailty.

| Variable | Hs-CTnT (≥14 vs. <14 ng/L) | | |  | NT-proBNP (≥100 vs. <100 ng/ml) | | |  | NT-proBNP (≥300 vs. <300 ng/ml) | | |
| --- | --- | --- | --- | --- | --- | --- | --- | --- | --- | --- | --- |
|  | HR (95% CI) | P | P for interaction |  | HR (95% CI) | P | P for interaction |  | HR (95% CI) | P | P for interaction |
| In total | 2.55 (1.51-4.28) | <0.001 | - |  | 1.67 (1.23-2.27) | 0.001 | - |  | 1.59 (0.80-3.15) | 0.184 | - |
| **Age** |  |  | 0.407 |  |  |  | 0.593 |  |  |  | 0.898 |
| <64 | 2.45 (1.34-4.48) | 0.004 |  |  | 1.48 (0.83-2.64) | 0.182 |  |  | 2.21 (0.63-7.72) | 0.215 |  |
| ≥64 | 2.15 (0.71-6.47) | 0.174 |  |  | 1.53 (1.06-2.23) | 0.025 |  |  | 1.26 (0.56-2.85) | 0.584 |  |
| **Gender** |  |  | 0.908 |  |  |  | 0.070 |  |  |  | 0.242 |
| Male | 2.60 (1.44-4.69) | 0.002 |  |  | 2.38 (1.41-4.01) | 0.001 |  |  | 0.41 (0.05-3.10) | 0.392 |  |
| Female | 2.12 (0.67-6.76) | 0.202 |  |  | 1.39 (0.95-2.04) | 0.084 |  |  | 2.34 (1.09-4.99) | 0.028 |  |
| **Race** |  |  | 0.968 |  |  |  | 0.271 |  |  |  | 0.998 |
| White | 2.44 (1.33-4.48) | 0.004 |  |  | 1.74 (1.21-2.49) | 0.003 |  |  | 1.74 (0.85-3.56) | 0.127 |  |
| Black | 2.33 (1.22-4.47) | 0.010 |  |  | 1.36 (0.72-2.58) | 0.343 |  |  | 1.04 (0.99-1.08) | 0.061 |  |
| **Hypertension** |  |  | 0.385 |  |  |  | 0.111 |  |  |  | 0.005 |
| No | 2.38 (1.45-3.91) | 0.001 |  |  | 1.49 (1.12-2.01) | 0.007 |  |  | 3.42 (2.15-5.43) | <0.001 |  |
| Yes | 1.78 (0.91-3.51) | 0.093 |  |  | 1.49 (0.97-2.31) | 0.066 |  |  | 1.04 (0.44-2.44) | 0.932 |  |
| **Diabetes** |  |  | 0.411 |  |  |  | 0.431 |  |  |  | 0.600 |
| No | 2.22 (1.40-3.51) | 0.001 |  |  | 1.58 (1.22-2.05) | 0.001 |  |  | 2.26 (1.42-3.49) | <0.001 |  |
| Yes | 1.60 (0.69-3.70) | 0.272 |  |  | 0.90 (0.46-1.76) | 0.763 |  |  | 1.88 (0.67-5.25) | 0.230 |  |

*The population was classified according to the median of age. Multivariate Cox regression analysis between cardiac biomarker and frailty adjusted by age, sex, center-race, education (<high school, high school, or >high school), smoking (never, former, current), drinking (never, former, current), body mass index, systolic blood pressure, heart rate, total cholesterol, triglycerides, cognition Z score, hypertension, diabetes, and cancer. hs-CTnT, high-sensitive cardiac troponin T; NT-proBNP, N-terminal pro-B-type natriuretic peptide. HR, hazard ratio; CI, confidence interval.
