# Supplementary figures and images for: Subclinical cardiovascular disease and frailty risk: the atherosclerosis risk in communities study
Source: BMC Geriatr. 2022 Apr 12;22:321. doi: 10.1186/s12877-022-02974-z (PMC9006603; doi:10.1186/s12877-022-02974-z)

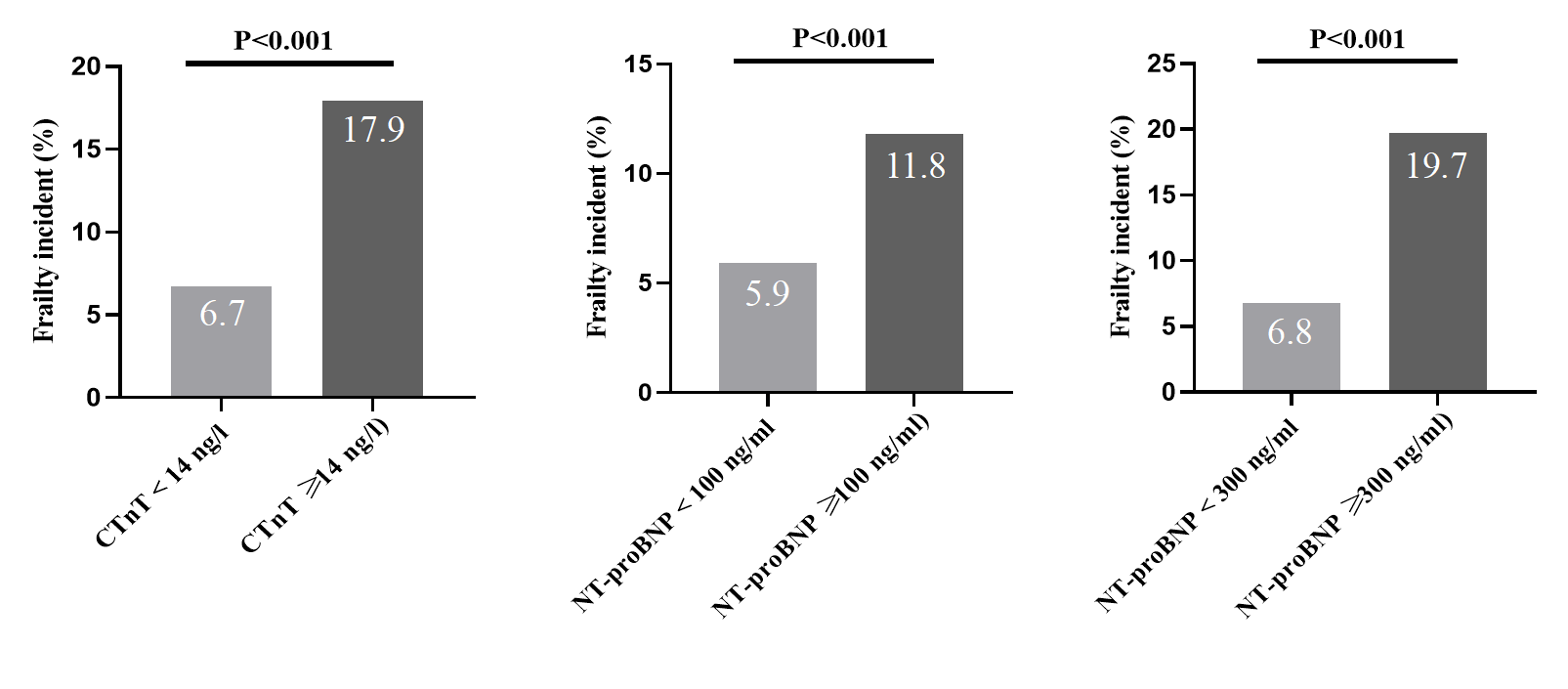

Supplement: Supplementary file 2 — Additional file 2. [file 12877_2022_2974_MOESM2_ESM.png]

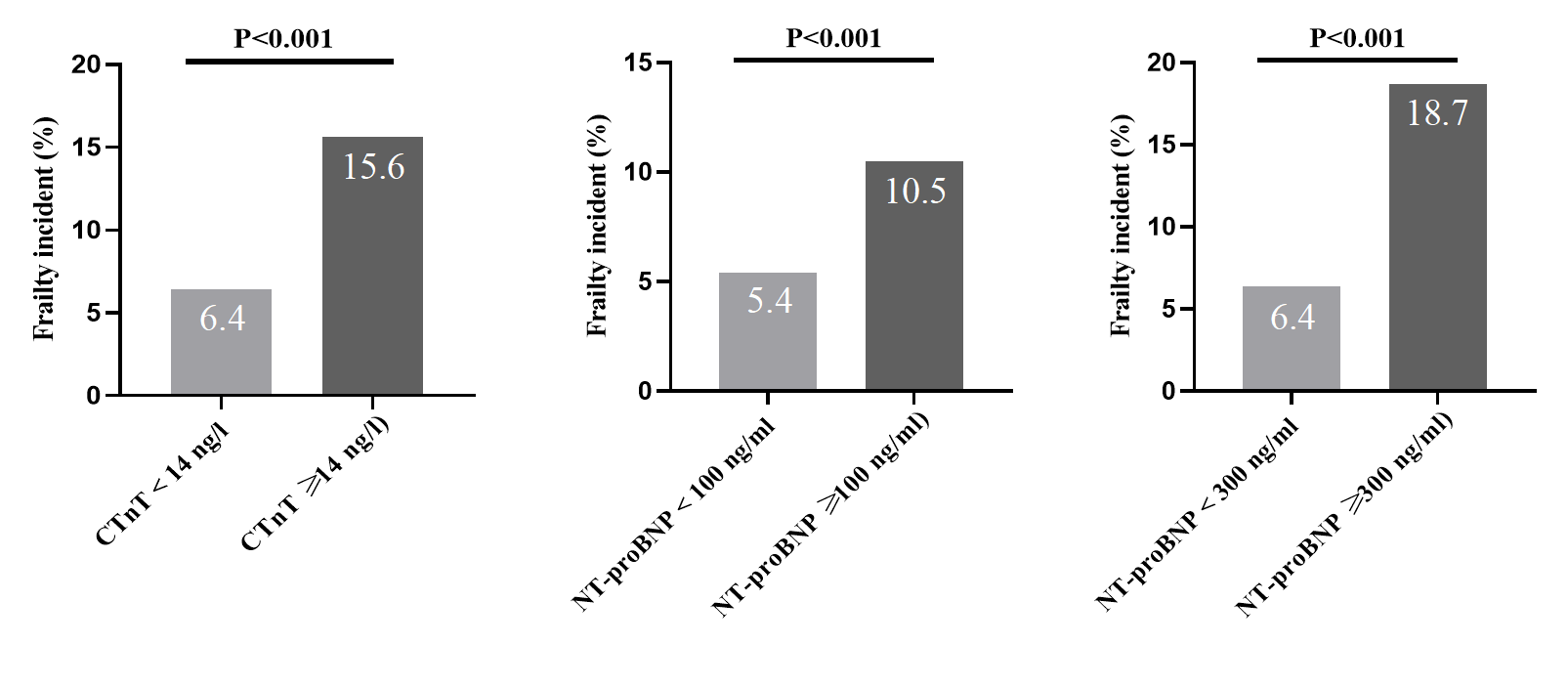

Supplement: Supplementary file 3 — Additional file 3. [file 12877_2022_2974_MOESM3_ESM.png]

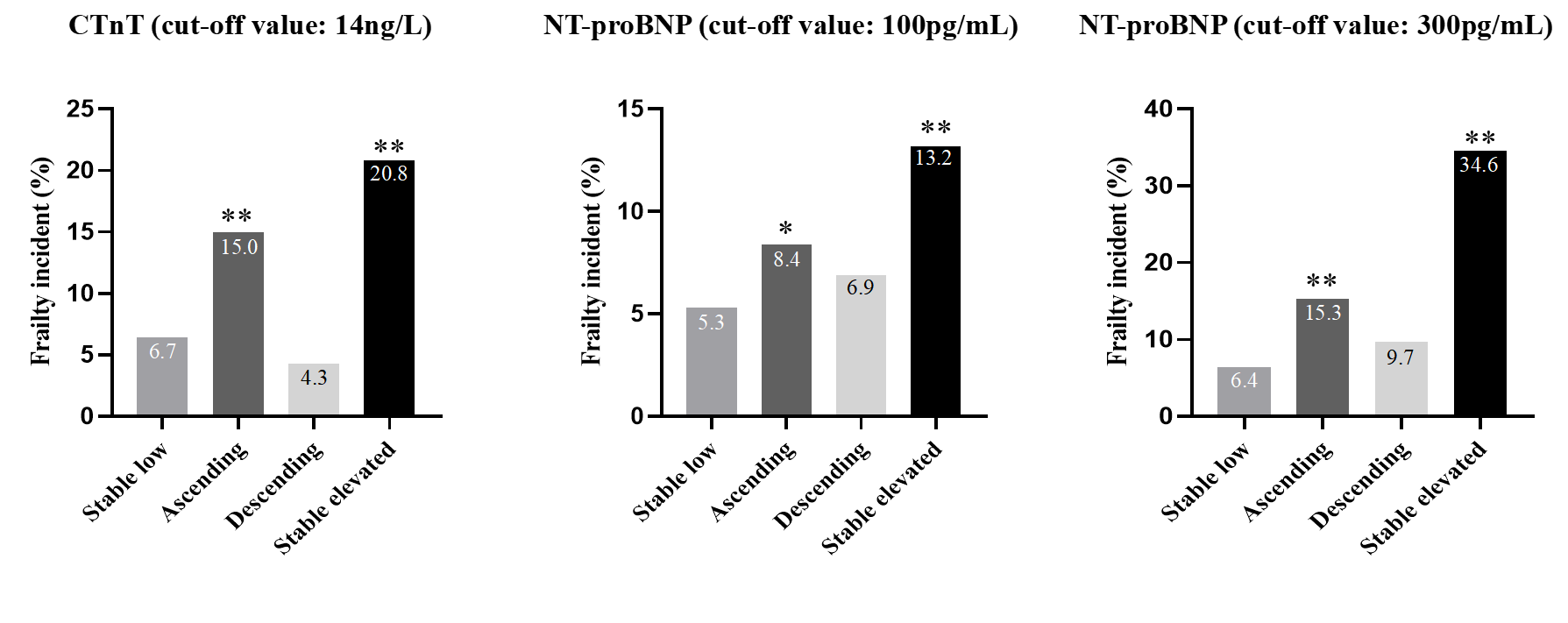

Supplement: Supplementary file 4 — Additional file 4. [file 12877_2022_2974_MOESM4_ESM.png]
